# Olefin metathesis of phospholipids by Ruthenium-based catalysts in solution and on liposomes under biologically relevant conditions

**DOI:** 10.1007/s00775-025-02129-6

**Published:** 2025-11-25

**Authors:** Pina Eichert, Huriye Deniz Uzun, Sascha Heinrich, Thomas Günther Pomorski, Nils Metzler-Nolte

**Affiliations:** 1 Faculty of Chemistry and Biochemistry, Inorganic Chemistry I – Bioinorganic Chemistry, Ruhr University Bochum, Bochum, Germany; 2 Faculty of Chemistry and Biochemistry, Molecular Biochemistry, Ruhr University Bochum, Bochum, Germany; 3 Department of Plant and Environmental Sciences, University of Copenhagen, Frederiksberg, Denmark; 4 Faculty of Chemistry and Biochemistry, Organic Chemistry I – Chemistry and Biochemistry of Natural Products, Ruhr University Bochum, Bochum, Germany; 5 Present Address: North-Rhine Westphalian State Agency for Nature, Environment, and Consumer Protection (LANUV NRW), Duisburg, Germany

**Keywords:** Bioorganometallic chemistry, Liposome, Olefin metathesis, Phospholipids, Ruthenium

## Abstract

The last decade has seen a lot of interest in classical organometallic catalysis performed intracellularly, or at least under biological conditions (37 °C, air, water). One such classical reaction is olefin metathesis (OM), which is extremely common in preparative organic chemistry, however under non-biological conditions (esp. organic solvents). For in vivo applications, both prokaryotic and eukaryotic applications would require the passage of the OM catalyst through the cell membrane, which already contains unsaturated olefins and thus potential substrates for OM. This work focuses on the question whether OM is catalysed in membranes containing unsaturated phospholipids. Initial experiments with the 2nd generation Hoveyda-Grubbs catalyst (**HGII**) showed self-metathesis of the membrane-forming phospholipids POPE, POPC, and POPG, while approximating biological conditions in solution (37 °C, air, neutral pH) with substrate conversions up to 58%. Subsequent experiments with DOPC in large unilamellar vesicles (LUVs) and in solution included a PEGylated (featuring increased water solubility) and a palmitoylated (with increased membrane mobility) **HGII** derivative. Both were successfully synthesised beforehand and comprehensively characterized. Membrane localisation of the catalysts was evaluated via size exclusion chromatography (SEC) followed by ICP-MS. Product analysis of OM was carried out after one hour and 24 h, respectively, via NMR, TLC, and LC-MS. The intra- and intermolecular OM products of DOPC were identified. First, substrate conversion was significantly diminished in the vesicles, however, the equilibrium state in vesicles was reached in much less time compared to solution (1 h in vesicles vs. 24 h in solution). To our surprise, a distinct difference in product selectivity between OM in solution and in vesicles was observed in that the intramolecular OM is much favoured in solution, while in LUVs, the main products are the result of intermolecular OM reactions. Our results prove that OM is readily catalysed in vesicles and indicate that the milieu of the lipid bilayer has a major impact on the product selectivity, reaction time and substrate conversion. While previous work has focused on the cytotoxicity of Hoveyda-Grubbs catalysts and their interaction with biomolecules, our present work provides valuable insights on what will happen when an OM catalyst like **HGII** is exposed to membranes in organisms. Moreover, using the results from this work, it may be possible to selectively modify membrane properties, and thereby cellular responsiveness to outside stress, by olefin metathesis in the future.

**Supplementary Information:**

The online version contains supplementary material available at 10.1007/s00775-025-02129-6.

## Introduction

Olefin metathesis (OM) is a versatile reaction for the rearrangement of carbon-carbon double bonds with different reaction types, e.g. ring-closing metathesis, cross metathesis, self-metathesis, ring-opening metathesis polymerisation or ethenolysis [1]. This reaction type has quickly found industrial applications, e.g. in the Shell Higher Olefin Process for the production of linear α-olefins or polymers [2, 3]. The general effort in preparative chemistry to find more sustainable and ecological synthesis strategies has put a focus on OM catalysis in aqueous media, leading to the development of modified catalysts with polyethylene glycol (PEG) or quaternary ammonium groups to increase water solubility and other strategies such as metathesis in emulsion or immobilisation of catalysts [4–7]. The perspective of olefin metathesis in aqueous media has been met with interdisciplinary curiosity from the fields of (chemical) biology and biochemistry. As a result, interest in biological applications or modifications of olefin metathesis has increased in recent years. A key factor is the remarkable stability of the 2nd generation’s ruthenium Hoveyda-Grubbs catalyst (**HGII**) towards water and oxygen compared to its predecessors, while remaining highly active even on inactivated olefins [8]. In addition, ruthenium offers a higher functional group tolerance towards esters, amides, alcohols, and acids than other transition metals, such as tungsten or molybdenum, used in olefin metathesis catalysis, which is a major advantage in the presence of biomolecules [1]. 

Ruthenium-based catalysts have been used for olefin metathesis in a number of biological applications. For example, artificial hybrid metalloenzyme have been constructed from a protein scaffold (host) and an HGII-type catalyst bound to the host either covalently or via an intermolecular bond [9–13]. Ward and co-workers published the first whole-cell assembly of a hybrid metalloenzyme for OM in 2016 [12]. Their construct took advantage of the biotin-streptavidin technology, with streptavidin secreted into the periplasm of bacteria and extracellularly added biotinylated HGII catalyst. Since then, other in vivo OM reactions have been reported, including the development of the “close-to-release” strategy, the catalysis of ring-closing metathesis in the periplasm of *Escherichia coli*, and the presence of HeLa cells by unmodified **HGII** [14]. Toussaint et al. demonstrated the use of metathesis catalysts as sensors for ethylene detection in *Chlamydomonas reinhardtii* [15]. With regard to possible toxicity, the bioactivity of Grubbs catalysts on human cancer cells was evaluated by Ott and his coworkers [16]. They reported overall morphological changes in cells, their membranes, and adhesions. Out of the derivatives tested, **HGII** showed the most distinct antiproliferative effects [16]. 

Every living cell, regardless of its nature or function, is encased in a cell membrane which acts as a protective boundary. This crucial membrane is primarily composed of different lipids, of which phospholipids are a major class. A number of phospholipids will feature olefins in their fatty acid chains, and double bonds are also present in other membrane components like cholesteryl or farnesyl moieties. Taking into consideration the hydrophobic character of **HGII**-type catalysts, the question emerges whether the catalyst might accumulate in the cell membrane and promote OM of those main membrane components. Consequently, the uptake of catalysts into living cells poses significant challenges and might result in significant conversions of unsaturated lipids. To the best of our knowledge, this question has not been experimentally considered so far. This work henceforth investigates the OM of membrane lipids, particularly phospholipids. We aim to evaluate the activity of **HGII**-type catalysts, which is already used for biological applications, to gain a deeper understanding of its interactions with biological membranes. In addition to the original **HGII** molecule, we synthesized two **HGII** conjugates: One with increased water solubility via PEGylation and another with enhanced membrane mobility via palmitoylation. These catalysts were compared in their reactivity towards saturated and unsaturated phospholipids with unmodified **HGII** both in solution and in liposome experiments.

## Results and discussion

### Synthesis of **HGII** derivatives

To elucidate the importance of catalyst properties in OM reactions with phospholipids, initially, two additional **HGII** derivatives were synthesised that are either water-soluble or contain a highly lipophilic membrane anchor.

**Scheme 1:**
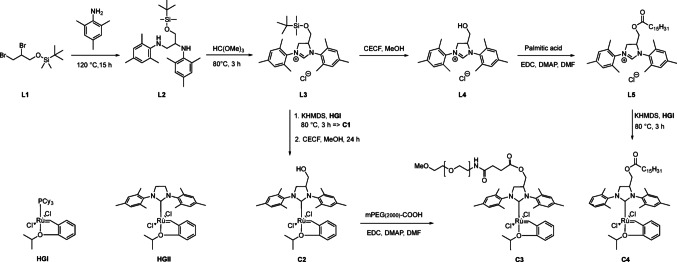
Synthesis route to catalysts **C3** and **C4** used in this work; **HGI**: 1^st^ generation Hoveyda-Grubbs catalyst; **HGII**: 2^nd^ generation Hoveyda-Grubbs catalyst. See expt. Section for details and suppl. material for data and spectra

The modified ruthenium catalysts, denoted **C1** - **C4**, were synthesised based on the hydroxymethyl N-heterocyclic carbene (NHC) ligand **L4** and its derivatives (Scheme 1). The NHC ligand synthesis was adapted from the literature with minor modifications in the synthesis sequence, temperature and time [17]. To access the PEGylated catalyst **C3**, its TBDMS-protected hydroxy-methylated precursor **C1** was obtained from the corresponding ligand **L1** and **HGI**. By stripping **C1** from the TBDMS-protecting group, the hydroxy-methylated precursor **C2** was generated allowing a *Steglich* esterification with mPEG(2000)COOH to synthesise **C3**. This approach is a deviation from the procedure published by Grubbs, in which mesylated mPEG is attached to the hydroxy NHC precursor via a S_N_2-type reaction and the PEGylated NHC ligand was synthesised prior to addition to the ruthenium centre of **HGI** [4]. After purification, **C3** was obtained with a yield of 68%. The ^1^H NMR spectrum showed, besides the signals for the NHC and benzylidene ligand, characteristic signals for Ru = C*H*(R) with a shift of 16.39 ppm and for PEG C*H*_2_ protons at 3.58 ppm with an integral of 219 Hs. MALDI-MS analysis complemented the results showing the typical PEG mass distributions and an average mass of *m/z* = 2417.5. *Steglich* esterification was used to obtain the palmitoylated NHC ligand **L5** from palmitic acid and **L4**. Catalyst **C4** was obtained in a moderate yield of 47% via adition of the **L5** carbene to the ruthenium centre of the precursor **HGI**. The ^1^H NMR spectra of **C4** confirmed the common ligand signals as well as the carbene proton in close vicinity to the ruthenium centre with a shift of 16.33 ppm. Characteristic signals for the palmitic acid moiety were also observed, with a triplet of the terminal methyl group at 0.80 ppm and the hydrocarbon chain observed at 1.18 ppm. An electrospray ionisation mass spectrometry (ESI-MS) spectrum revealed the isotopic distribution pattern for ruthenium and the correct mass of 859.4 *m/z* (calculated: [M-Cl]^+^ = 860.42 *m/z*).

### Metathesis of phospholipids with the unmodified catalyst **HGII**

**Fig.1:**
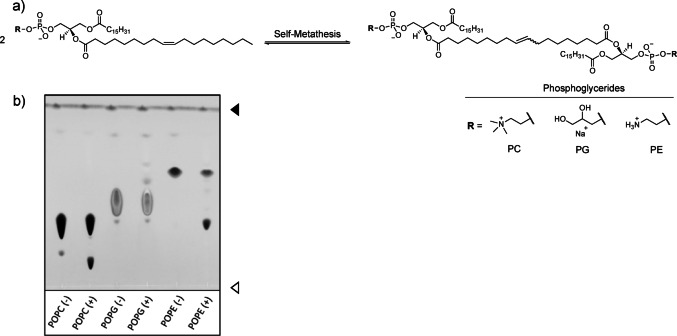
Olefin metathesis of various phospholipids in solution (MeOH, 5 mol% catalyst, 37 °C). (**a**) Structures of unsaturated phospholipids POPC, POPE, and POPG as well as general self-metathesis reaction scheme of POPC, POPE, and POPG. (**b**) Representative thin-layer chromatography of metathesis reactions without (-) and with (+) catalyst **HGII** after 24 h. Lipids were visualized by staining with primulin. Open arrow, origin; filled arrow, solvent front of the chromatograms

Initial metathesis tests on phospholipids aimed to establish the general activity of the unmodified catalyst HGII in solution. Given HGII’s insolubility in water, methanol was employed as a surrogate solvent due to its protic nature and its ability to dissolve both the catalyst and substrate. Phospholipids featuring palmitic acid and oleic acid at the *sn-1* and *sn-2* positions were chosen, ensuring the exclusive formation of a singular metathesis product (Fig. 1a). After 24 h incubation, the reaction mixtures were analysed by thin-layer chromatography (TLC, Fig. 1b). The major metathesis products were then identified by ESI-MS. In addition, the metathesis products of phosphatidylethanolamine (POPE) and phosphatidylcholine (POPC) were isolated and characterised by proton nuclear magnetic resonance spectroscopy (^1^H NMR). The analysis showed that HGII effectively catalysed OM of monounsaturated phospholipids, yielding the expected short dimeric phospholipids. Notably, the conversion rates varied for the different phospholipids as shown in Table 1. Specifically, phosphatidylglycerol (POPG) showed the lowest conversion (15.5%) while phosphatidylethanolamine (POPE) had the highest conversion (57%). Phosphatidylcholine (POPC) showed an intermediate conversion (23%). As a negative control, saturated dilauroyl-phosphatidylcholine (DLPC) was used and recovered unchanged, thus confirming the expected absence of product or adduct formation (Supplementary Figure S1).

Generally, OM in solution is assumed to be in equilibrium in the absence of perturbing factors. Therefore, a lipid conversion of 50% would be anticipated in the absence of other favourable factors. Previous studies on the OM of unsaturated fatty acids, esters, or lipids have primarily focused on industrial applications [3, 18, 19], employing conditions such as thermodynamic sinks, elevated temperatures, aprotic organic solvents, and neat solutions to achieve high substrate conversions up to 100% with 0.1 mol% catalyst [20–25]. However, these conditions are not applicable for estimating the extent of OM under biological conditions. Our experiments were conducted under different conditions, lacking strong driving forces, and therefore, substrate conversions well above 50% were neither expected nor achieved.

Possible explanations for the observed differences in conversion rates need to include the unique properties of the different phospholipids tested. POPE can form both hydrogen bonds and ion-ion interactions, fostering stable interactions and thus proximity between two POPE molecules. In contrast, POPC lacks proton-donating and accepting properties, and its quaternary amino group is more shielded, potentially reducing its conversion. POPG can form intermolecular hydrogen bonds but carries a negative charge, which may result in the repulsion of a second POPG molecule, accounting for the low conversion rate observed.

**Table 1:** Overview of the detected self-metathesis (SM) products of different unsaturated phospholipids (MeOH, 5 mol% catalyst, 37 °C). MS data were obtained via ESI-MS

Phospholipid	SM product	Substrate conversion (%]	Product mass [m/z] (Calculated)
POPG	**1**	15.5	[M-2Na]^2–^ = 621.8 (621.4)
POPC	**2**	23	[M + H]^+^ = 1267.4 (1267.9) [M + Na]^+^ = 1289.2 (1289.9)
POPE	**3**	57	[M + Na]^+^ = 1205.5 (1205.8)

### Metathesis of phospholipids with functionalised catalysts

Next, we conducted a series of experiments using the two functionalised OM catalysts **C3** (PEGylated) and **C4** (palmitoylated) alongside unmodified **HGII** with the di-unsaturated DOPC as substrate. DOPC was chosen because it is used in subsequent liposome preparations and to investigate product formation with di-unsaturated lipids. Reactions were performed in methanol for 1 h and 24 h, respectively. TLC analysis showed product formation after one hour for all three catalysts, with significant variations in activity (Fig. 3a, c).

**Fig. 2:**
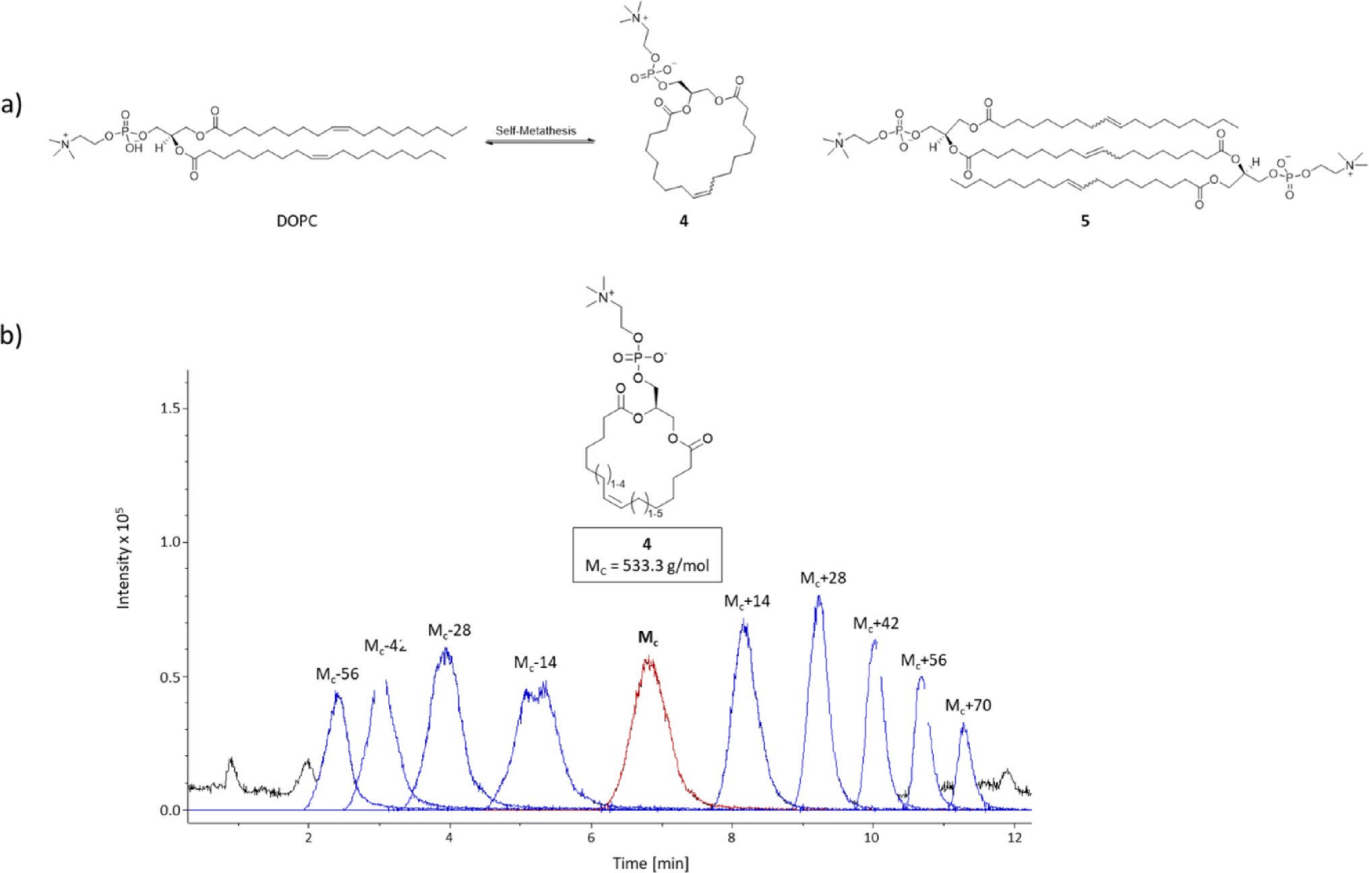
Self-metathesis of DOPC with catalyst **HGII** according to the preparative procedure (5 mol% catalyst, 37 °C). (**a**) Scheme of self-metathesis of DOPC with the expected products **4** (intramolecular) and **5** (intermolecular). (**b**) Chromatogram of the LC-MS analysis of the intramolecular product **4** demonstrating double bond migration via variations of the ring size, M_C_ (red) marks the peak of **4** with the molecular mass M_C_ = 533.3 g/mol without any isomerisation of the double bond. The residual peaks (blue) are assigned to the derivatives of **4** with the mass deviation of M_c_ (e.g. M_c_ + 14 represents the **4** derivative with one additional methyl group) given above the corresponding peak. See also Table S1

**Fig. 3:**
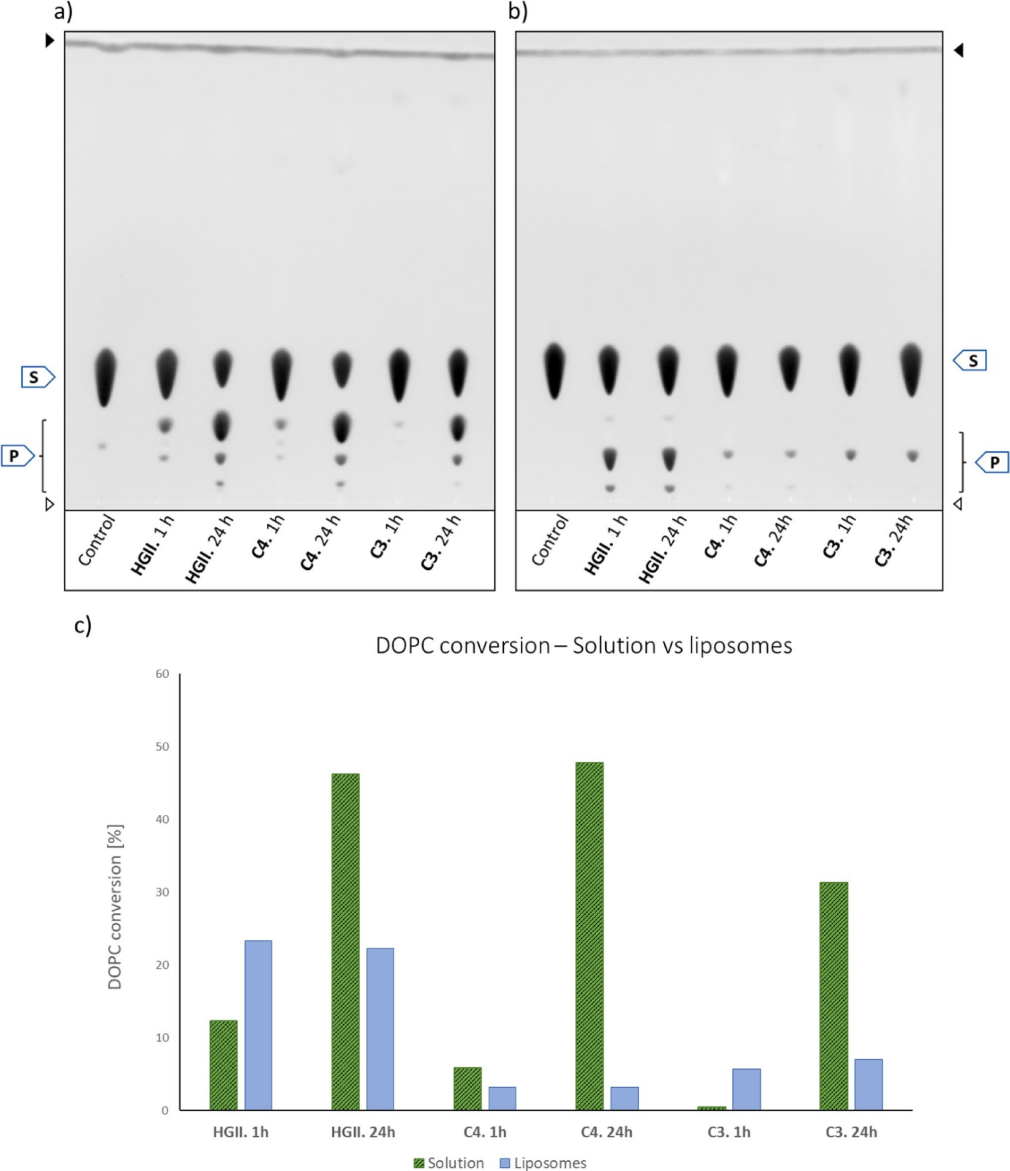
Self-metathesis of DOPC with catalysts **HGII**, **C3**, and **C4** in solution (MeOH, 37 °C, 5 mol%) and liposomes (200 nm, in HEPES buffer, pH 7.4, 37 °C, 5 mol%). (**a**) Thin-layer chromatography (TLC) of lipid extracts from the metathesis reaction in solution. (**b**) TLC of lipid extracts from metathesis reaction in liposomes (LUVs). Lipids were visualized by primuline staining. S, substrate (DOPC); P, products. Open arrow, origin; filled arrow, solvent front of the chromatograms. (**c**) DOPC conversions in the presence of **HGII**, **C3**, and **C4** for each metathesis reaction in solution (green) and in liposomes (blue) for the indicated times, see expt. section for details

After 1 h, the **HGII** reaction mixture showed two product spots with distinct intensity and a DOPC conversion of 12.3%, whereas **C4** showed three products with low intensities and a DOPC conversion of 5.8% (Fig. 3a, c). The PEGylated catalyst **C3** showed the lowest performance after 1 h, with only a trace amount of one product spot and 0.5% DOPC conversion. After a reaction time of 24 h, it can be assumed that an equilibrium state has been reached. Catalysis with **HGII** and **C4** reached similar conversions of 46.2% and 47.8% respectively, whereas **C3** displayed lower activity with a conversion of 31.3%. A total of three metathesis product spots were observed next to the DOPC spot (R_f_ = 0.3). Figure 2a depicts a general scheme of the self-metathesis of DOPC, including both intramolecular (**4**) and intermolecular (**5**) DOPC products, together with the LC-MS chromatogram. Product **4** was identified as the intramolecular cyclization product via ^1^H NMR spectroscopy and mass spectrometry, had an R_f_ value of 0.18 and was formed by all three catalysts (Fig. 3a). ^1^H NMR analysis revealed signals and shifts similar to those of DOPC, but with broader signals and without the terminal methyl group, thereby indicating complete conversion of the double bond by OM. For product identification, the ratio between olefinic protons and the proton on the chiral carbon proved to be the most conclusive. While in DOPC the ratio is 4:1, product **4** had a ratio of 2:1, corresponding to one double bond per phosphatidylglycerol. This was supported by initial ESI-MS data, which provided the mass of the corresponding protonated molecule (M_c_ = 533 *m/z*) as well as the sodium adduct. However, further LC-MS analysis of **4** revealed the formation of different products with a mass variation of multiples (n) of 14, ranging from *m/z* = M_C_ – 56 (*n* = − 4) to *m/z* = M_c_ +70 (n = + 5). This suggests that **4** is actually a mixture of products that vary in ring size and can be (multiples of) one methylene group smaller and larger than the calculated mass M_c_ (Fig. 2b). In consideration of the NMR data, which overall showed the presence of all common lipid signals, we conclude that this variation is the result of double bond isomerization. This isomerization shifts the double bond by one methylene group (CH_2_, m = 14) along the fatty acid chain and occurs *before* OM, ultimately resulting in slightly smaller or larger ring size in **4**. It should be noted that such double bond isomerization is commonly catalysed by Grubbs catalysts [26, 27]. 

Product **5** (R_f_ = 0.10) was identified as the dimeric product formed only by **HGII** and in traces by **C4** after one hour. The ^1^H NMR spectrum showed shifts and integrals even more similar to DOPC, but the signals appeared to be broader again. The proton ratio between olefinic and chiral position was 3:1, indicating a structure with three double bonds shared between two phosphatidylglycerol head groups. This was supported by ESI-MS data, which revealed the mass of the corresponding protonated molecule as well as its sodium adduct. It is noteworthy that the integrals in the ^1^H NMR spectra are not completely consistent/congruent with the calculated proton count. This might be a consequence of double bond migration occurring prior to OM, as described above for **4**. As a consequence, the various isomerisation products are unlikely to be separated by chromatography. Product **6** (R_f_ = 0.05), which appeared after 24 h in reaction mixtures with **HGII** and **C4**, was visible as a trace in the sample containing **C3**. Product **6** has not yet been identified. Based on the low R_f_ value and the possibility of olefin metathesis of three DOPC molecules, it is assumed that product **6** is an extended derivative of **5**. The OM catalysed by **HGII** and **C4** are in an equal equilibrium state, for which a conversion close to 50% is the expected conversion rate, based on our previous experiments. The PEGylated **C3** displayed a lower performance than the other catalysts. This could be due to the long PEG chain, blocking or shielding the catalyst, or some other effect which was not further investigated in the current study.

## Metathesis in DOPC liposomes

To investigate the metathesis of membrane-embedded phospholipids, experiments were carried out using liposomes prepared from DOPC as a model membrane system. DOPC liposomes were prepared as large unilamellar vesicles (LUVs) in HEPES buffer and catalysts **HGII**, **C3** and **C4** were added from a stock solution in methanol. Characterizing data for liposomes are presented in the suppl. material. Olefin metathesis was performed for 1 and 24 h, as before. TLC analysis of the samples in liposomes revealed two major differences compared to the previous experiment in methanol solution (compare Figs. 3a and b). First, the sample lanes between 1 and 24 h showed a striking similarity for each catalyst. This suggests that the olefin metathesis in the vesicles reaches its equilibrium state already after 1 h irrespective of the catalyst used and thereby is much faster than in solution. The DOPC conversion by **HGII** reached 23.3 and 22.3% after 1 and 24 h, respectively, which is almost twice as much as in solution over the same time interval (Fig. 3c). Conversely, the lowest DOPC conversion over time was observed for **C4**, reaching 3.2% after 1 and 24 h. In comparison, **C3** showed a slight increase in activity, with a DOPC conversion of 5.7% after 1 and 7.0% after 1 and 24 h, respectively.

It is noteworthy that **C4** showed the lowest activity on liposomes, but the highest activity in solution of all the catalysts tested, whereas **HGII** had an activity close to **C4** in solution and the highest activity on liposomes. Since the experiments in solution showed a significantly higher activity of both **C3** and **C4**, it is most likely that both catalysts are less efficiently inserted into the membrane, despite their different functionalization. This notion is in line with the observed low liposomes association of the catalysts (Suppl. Table S2). In general, the DOPC conversion in liposomes is significantly diminished compared to the same reaction in solution. The main reason for this is likely the membrane barrier, meaning that the catalysts have to pass the polar membrane surface first.

Secondly, a remarkable difference in product distribution is visible in the TLCs as the spot originating from compound **5** is the most intense for samples with **HGII** and **C4** and the sole product for samples with **C3**. Samples with **HGII** show spots for products **4** to **6**, however, the spot for **4** is only visible as a trace, unlike the equivalent product spot in methanol. Compounds **5** and **6** showed distinct intensities. Catalyst **C3** and **C4** showed a similar performance with **5** being the main product, while **6** is only visible as a trace for **C4**. In liposomes, **5** is the main product for all catalysts, whereas in solution **4** is preferred.

The observed product selectivity could be a result of the different lipid order. A homogeneous solution of lipids offers increasing probabilities for unconverted DOPC molecules to collide with the catalyst through Brownian motion. Due to the dilution, intramolecular self-metathesis (SM) is favoured, yielding **4** as the main product. On the other hand, the lipid bilayer of liposomes provides a preorganised system, in which hydrocarbon chains are stacked and obviously highly concentrated compared to solution. The catalyst is exposed to this preorganisation and is more likely to rearrange carbon atoms from two different DOPC molecules, resulting in **5** as the main product. Although the membrane is a fluid system, it seems likely that the catalyst cannot move as far (or fast) upon membrane insertion and thus reacts more often with products than with unconverted DOPC. This explains why the equilibrium state is achieved already after one hour in liposomes, while DOPC conversion increases in solution over a longer period.

Naturally, different yields for OM could also be due to different amounts of catalyst. In order to probe the ruthenium content in the liposomes contributed by the different Ru catalysts, DOPC-based liposomes incubated with **HGII**, **C3**, and **C4** were purified via SEC, and the ruthenium content of the eluates was determined via ICP-MS. Before SEC, the LUVs incubated with catalysts displayed ^102^Ru concentrations in a similar range, 3347.3 ppb (LUV + **HGII**) to 4652.6 ppb (LUV + **C3**). The palmitoylated catalyst **C4** was the least soluble in the methanol/water mixture that was used for sample preparation and partially precipitated. Therefore, the results of **C4** were considered unreliable and discarded. After SEC, similar ^102^Ru concentrations were measured for the liposome samples with **HGII** and **C3** (1629.1 ppb and 1523.3 ppb).

One third of the ruthenium from **HGII** was eluted from SEC, which seems to indicate that only a third of the added **HGII** was effectively incorporated into the liposomes or at least strongly associated to the liposomal membrane. However, we also need to consider that the total amount of liposomes recovered from SEC is unknown. Therefore, the obtained data are not to be considered as absolute values, but rather a lower limit. The reduced ruthenium content measured for **C3** compared to **HGII** is in line with the lower DOPC conversion in liposomes with 7%. It was anticipated that the water-soluble complex **C3** shows amphiphilic characteristics, allowing a higher membrane integration of the lipophilic catalytic moiety. In comparison to the unmodified **HGII**, these expectations are not supported by our results. It might be possible that the PEG chain (2 kDa) was too long and thus shielding the catalyst or might induce micelle formation by itself.

## Conclusion

We investigated OM of mono- and di-unsaturated phospholipids in solution and for the first time also on liposomes with the 2nd generation Hoveyda-Grubbs catalyst (**HGII**) and two derivatives, a water soluble PEGylated **HGII** (**C3**) and a palmitoylated **HGII** derivative (**C4**). Our studies show that **HGII** catalyses the self-metathesis of phospholipids in solution under conditions close to a biological environment (37 °C, neutral pH, protic solvent) with substrate conversions between 15.5 and 57% after 24 h. Under these conditions, OM of these substrates is an equilibrium reaction, giving 50% conversion as maximum in the absence of any other thermodynamic or kinetic driving force. The successful OM of mono-unsaturated phospholipids like POPC, POPE, and POPG under the restricting conditions used herein represents a successful proof of principle. Further experiments on the di-unsaturated DOPC included **C3** and **C4** and showed a similar range of substrate conversion (31–48%), confirming the initial experiments for all tested catalysts in solution. TLC analysis showed three distinct product spots with the intramolecular cyclization product (**4**) as the dominant species, followed by the self-metathesis product (**5**). The third product remained unidentified but is believed to be a larger phospholipid possibly with four double bonds and three phosphate groups. Additionally, DOPC-based liposomes (*l*arge *u*nilamellar *v*esicles, LUVs) were used as model membrane systems, and OM was also explored in these systems. Overall liposomes showed lower DOPC conversion compared to solution (3% – 23%). The conversions for **C3** and **C4** were in a similar range, below 7%, whereas **HGII** was significantly more active (23%). This could be a result of reduced membrane transition of the catalysts. Indeed, ICP-MS data indicate membrane association of **HGII** and **C3**. Most surprising, however, was the difference in product selectivity which was observed between OM in solution and in model membranes. While the cyclic, *intra*molecular DOPC metathesis product **4** was preferentially formed in solution, the *inter*molecular DOPC product **5** was favoured in vesicles. This may well be due to the dense packing and preorganisation of the hydrocarbon chains in the lipid bilayer of the liposomes, which allows the catalyst to easily interact with fatty acid residues of *two separate* molecules, whereas in solution the lipids will be more dilute at the same concentration and intramolecular OM is far more likely. It is also noteworthy that the substrate conversion barely increased any further after one hour in LUVs, whereas in solution most of the conversion was achieved after reaction times much longer than one hour. This suggests that the reaction reaches an equilibrium state faster in the vesicles than in solution but at the cost of product yield. Taken together, this could be an indication of possible interactions between an OM catalyst such as **HGII** and the cell membrane. In exploring the bio-applicability of **HGII**-type catalysts, previous work has mainly focused on cytotoxicity and the presence of potentially inhibiting biomolecules, such as those containing thiol groups [16, 28]. Our current work shows that it is also crucial to consider all biomolecules and structures, and focuses on potential substrates that the catalyst encounters upon entering a cell.

## Experimental

### Materials and Methods

All syntheses were carried out under *Schlenk *conditions in an Argon atmosphere unless mentioned otherwise. Solvents were dried over molecular sieve. All chemical compounds and solvents were purchased in analytical grade from Sigma Aldrich, TCI, VWR and Carbolution. Phospholipids 1,2-dilauroyl-phosphatidylcholine (DLPC), 1-palmitoyl-2-oleoyl-phosphatidylcholine (POPC), 1,2-dioleoyl-phosphatidylcholine (DOPC), -phosphatidylglycerol (DOPG), and -phosphatidylethanolamine (DOPE) were purchased from Avanti Polar Lipids Inc. (Alabaster, AL, USA). Large unilamellar vesicles (LUV) were prepared using a mini extruder from Avanti Polar Lipids with a 100 nm polycarbonate membrane provided by Whatman. NMR spectra were recorded on Bruker AVIII-300 and DRX-400. Figures S3 – S6 in the suppl. Material show NMR spectra. MS data were measured via electrospray ionisation (ESI) on a Bruker Esquire 6000 spectrometer. ICP-MS data were generated on a Thermo Scientific iCAP RQ. LC-MS analysis of the DOPC metathesis products were carried out on a Vion IMS QTof system with an EC 150/2 Nucleodur C18 ISIS column.

### Synthesis

#### 1-(tert-Butyldimethylsilyl)−2,3-dibromopropane,**L1**

A 100 mL round bottom Schlenk flask was equipped with 13 mL dry DMF. 2,3-Dibromopropanol (8 mL, 77.8 mmol) was added to the DMF. Imidazol (2 eq, 10.6 g, 155.7 mmol) and tert-butyldimethyl silyl chloride (11.73 g, 77.8 mmol) were added gradually. The reaction mixture was stirred at room temperature for 20 h. The reaction was quenched by adding 100 mL distilled water. The aqueous phase was extracted four times with 120 mL diethyl ether each. The combined organic layers were dried over MgSO_4_, filtered and concentrated de vacuo. The crude product mixture was distilled under reduced pressure. The impurities were removed at 58 °C and 1.2 mbar leaving a colourless oil as a pure product (17.5 g, 68 %). ^1^H-NMR (300 MHz, CDCl_3_): δ = 4.17 (m, 1H, C*H*_2_OSi), 4.03 (dd, 1H, C*H*HBr), 3.89 (m, 1H, C*H*_2_Osi), 3.83-3.73.83.73 (m, 2H, C*H*_2_Br), 0.91 (s, 9H, Si(C(C*H*_3_)_3_), 0.09 (s, 6H, Si(C*H*_3_)_2_) ppm.

#### 3-((tert-butyldimethylsilyl)oxy)-N^1^,N^2^-dimesitylpropane-1,2-diamine,**L2**

A 100 mL round bottom *Schlenk* flask was equipped with 13 mL dry DMF. 2,3-Dibromopropanol (8 mL, 77.8 mmol) was added to the DMF. Imidazol (2 eq, 10.6 g, 155.7 mmol) and tert-butyldimethyl silyl chloride (11.73 g, 77.8 mmol) were added gradually. The reaction mixture was stirred at room temperature for 20 h. The reaction was quenched by adding 100 mL distilled water. The aqueous phase was extracted four times with 120 mL diethyl ether each. The combined organic layers were dried over MgSO_4_, filtered and concentrated de vacuo. The crude product mixture was distilled under reduced pressure. The impurities were removed at 58 °C and 1.2 mbar leaving a colourless oil as a pure product (17.5 g, 68 %). ^1^H-NMR (300 MHz, CDCl_3_): δ = 4.17 (m, 1H, C*H*_2_OSi), 4.03 (dd, 1H, C*H*HBr), 3.89 (m, 1H, C*H*_2_Osi), 3.83-3.73.83.73 (m, 2H, C*H*_2_Br), 0.91 (s, 9H, Si(C(C*H*_3_)_3_), 0.09 (s, 6H, Si(C*H*_3_)_2_) ppm.

#### 3-((tert-butyldimethylsilyl)oxy)-N^1^,N^2^-dimesitylpropane-1,2-diamine,**L2**

A reaction mixture of **L1** (16.5 g, 50 mmol), 2,4,6-Trimethylaniline (3 eq., 21.1 mL, 150 mmol) and DiPEA (8.6 mL, 50 mmol) was prepared in a 100 mL round bottom *Schlenk* flask. The mixture was stirred at 135 °C for 15 h. After cooling down to room temperature, 50 mL DCM were added. The organic phase was washed four times with 60 mL 6M HCl each and twice with distilled water. The dried organic phase was purified via flash chromatography with n-hexane and ethyl acetate. The pure product was obtained as a light brown oil (8.59 g, 39 %). ^1^H-NMR (300 MHz, CDCl_3_): δ = 6.84 (s, 4H, *H*_Ar_), 3.76 (dd, 1H, C*H*_2_OSi), 3.68 (dd, 1H, C*H*_2_OSi), 3.52 (m, 1H, C*H*NH), 3.32 (dd, 1H, C*H*_2_NH), 2.94 (dd, 1H, C*H*_2_NH), 2.30 (s, 6H, *p*C*H*_3_), 2.28 (s, 6H, *o*C*H*_3_), 2.24 (s, 6H, *o*C*H*_3_), 0.92 (s, 9H, Si(C(C*H*_3_)_3_), 0.06 (d, 6H, Si(C*H*_3_)_2_) ppm. ESI-MS: [M+H]^+^= 441.3 *m/z*, [M+Na]^+^=463.2 *m/z*

#### 5-(((tert-butyldimethylsilyl)oxy)methyl)−1,3-dimesityl-4,5-dihydro-1H-imidazol-3-ium chloride, **L3**

**L2** (652.1 mg, 1.48 mmol) was dissolved in trimethylorthoformate (20 eq, 3.24 mL, 29.6 mmol) in a 25 mL round bottom *Schlenk* flask. Then, concentrated HCl (1.14 eq, 141 µL) was added and the reaction mixture was stirred at 80 °C for 3.5 h. The solvent was removed at 40 °C via an external cooling trap yielding a white precipitate. The crude product was stirred in diethyl ether at room temperature for 30 min and washed with toluene (40 °C), diethyl ether and pentane. A clean white powder was obtained (596.5 mg, 83 %). ^1^H-NMR (300 MHz, CDCl_3_): δ = 9.33 (s, 1H, NC*H*N), 6.85 (s, 4H, *H*_Ar_), 5.26 (t, 1H, NCH_2_C*H*N), 4.74 (t, 1H, NC*H*_2_CHN), 4.18 (t, 1H NC*H*_2_CHN), 3.77 (d, 1H, NC*H*_2_O), 3.62 (d, 1H NC*H*_2_O), 2.38 (s, 3H, *p*C*H*_3_), 2.31 (d, 9H, *o/p*C*H*_3_), 2.20 (s, 6H, *o*C*H*_3_), 0.80 (s, 9H, Si(C(C*H*_3_)_3_), −0.01 (d, 6H, Si((C*H*_3_)_2_) ppm. ESI-MS: [M-Cl]^+^=452.1 *m/z*

#### 5-(Hydroxymethyl)−1,3-dimesityl-4,5-dihydro-1H-imidazol-3-ium chloride,**L4**

The precursor **L3** (107.8 mg, 0.22 mmol) was dissolved in 1 mL dry methanol in a *Schlenk* tube. 1-Chloroethyl chloroformate (2 eq, 47.7 µL, 0.44 mmol) was added slowly dropwise. After a reaction time of 24 h at room temperature the solvent was removed via vacuum. The white precipitate was washed with diethyl ether and pentane. A white powder was obtained (78.6 mg, 95 %). ^1^H-NMR (300 MHz, CDCl3): δ = 7.91 (s, 1H, NC*H*N), 6.97 (m, 4H, *H*_Ar_), 5.15 (m, 1H, NCH_2_C*H*N), 4.82 (m, 1H, NC*H*_2_CHN), 4.40 (t, 1H, NC*H*_2_CHN), 3.96 (d, 1H, NC*H*_2_O), 3.55 (d, 1H, NC*H*_2_O), 2.48 (s, 6H, *o*C*H*_3_), 2.36 (s, 3H, *p*C*H*_3_), 2.30 (d, 9H, *o/p*C*H*_3_) ppm. ESI-MS: [M-Cl]^+^=337.1*m/z*

#### 1,3-Dimesityl-5-((palmitoyloxy)methyl)−4,5-dihydro-1H-imidazol-3-ium, **L5**

Palmitic acid (2 eq, 69.6 mg, 0.27 mmol) was dissolved in 3 mL dry DMF and cooled in an ice bath. EDC hydrochloride (3 eq, 78.2 mg, 0.41 mmol) was added and the mixture was stirred for 30 min. DMAP (0.3 eq, 5.2 mg, 0.042 mmol) was added. After 30 min **L4** (50.6 mg, 0.14 mmol) was added. The ice bath was removed and the reaction was carried out at room temperature for 18 h. The crude product was purified via flash chromatography with DCM and methanol (29.5 mg, 35 %). ^1^H-NMR (300 MHz, CDCl_3_): δ = 9.80 (s, 1H, NC*H*N), 6.76 (s, 4H, C_Mes_*H*), 5.30 (m, 1H, NCH_2_C*H*N), 4.76 (t, 1H,), 4.19 (dd, 1H,), 3.96 (m, 1H), 3.87 (dd, 1H), 2.26-2.11.26.11 (m, 18H, C_Mes_C*H*_3_), 1.44 (m, 2H, C(O)C*H*_2_), 1.16 (s, 27H, CH_2_), 0.77 (t, 3H, C*H*_3_) ppm. ESI-MS: [M-Cl]^+^=575.4 *m/z*

#### (4(((tert-Butyldimethylsilyl)oxy)methyl)−1,3-dimesitylimidazolidin-2-yl) (2-isopropoxybenzylidene) ruthenium(II)dichloride, **C1**

The ligand **L4** (1.7 eq, 500 mg, 1.03 mmol) and KHMDS (2 eq, 241 mg, 1.21 mmol) were dissolved in 12 mL dry toluene and stirred for 1 h. A solution of Hoveyda-Grubbs catalyst of the 1^st^ generation (362.6 mg, 0.6 mmol) in 15 mL toluene was added to the first solution. Amberlyst-15 was added and the reaction was carried out at 80 °C for 3 h. The filtered reaction mixture was purified three times via flash chromatography with n-hexane and ethyl acetate. The catalyst **1** was obtained as a green solid (242.4 mg, 52 %). ^1^H-NMR (300 MHz, CD_2_Cl_2_): δ = 16.45 (s, 1H, Ru=C*H*), 7.55 (m, 1H, H_Ar_), 7.06 (m, 4H, C*H*_Mes_), 6.97-6.82.97.82 (m, 3H, H_Ar_), 4.87 (sept, 1H, C*H*(CH_3_)_2_) 4.50 (m, 1H), 4.24 (t, 1H,), 4.05 (t, 1H), 3.78 (m, 2H), 2.41 (m, 18H, C_Mes_C*H*_3_), 1.22 (m, 6H, CH(C*H*_3_)_2_), 0.85 (s, 9H, Si(C(C*H*_3_)_3_), 0.03 (d, 4H, Si(C*H*_3_)_2_) ppm. ESI-MS: [M-Cl]^+^= 734.9 *m/z*

#### (4-Hydroxymethyl)−1,3-dimesitylimidazolidin-2-yl)(isopropoxybenzylidene)ruthenium(II) dichloride, **C2**

A 5 mL round bottom Schlenk flask was equipped with 2 mL dry methanol and **1** (121.2 mg, 0.16 mmol). 1-Chloroethyl chloroformate (0.5 eq, 8.5 µL, 0.079 mmol) was added slowly. The mixture was stirred at room temperature for 20 h and purified twice via flash chromatography with DCM and methanol. A green, pure solid was obtained (60.8 mg, 59 %). ^1^H-NMR (300 MHz, CD_2_Cl_2_): δ = 16.45 (s, 1H, Ru=C*H*), 7.54 (t, 1H, H_Ar_), 7.07 (d, 4H, C_Mes_*H*), 6.92 (m, 2H, H_Ar_), 6.83 (d, 1H, H_Ar_), 4.89 (sep, 1H,), 4.27 (t, 1H,), 4.07 (m, 1H,), 3.78 (m, 1H,), 3.62 (m, 1H,), 2.42 (br, 18H, C_Mes_(C*H*_3_)), 1.26 (m, 6H, CH(CH3)2) ppm. ^13^C-NMR (100 MHz, CD_2_Cl_2_): δ = 296.8 (Ru=*C*), 152.4 (N*C*N), 145.4 (^i^Pr-O-*C*_Ar_), 139.4 (*C*_Mes_, C_Ar_), 130.4 (*C*_Mes_), 130.1 (*C*_Mes_), 129.7 (*C*_Mes_, C_Ar_), 122.7 (*C*_Ar_), 122.6 (*C*_Ar_), 113.4 (CH-*C*_Ar_), 75.5 (NCH_2_*C*HN& *C*H(CH_3_)_2_), 65.9 (*C*H_2_OH), 63.2 (N*C*H_2_CHN), 21.4 (C_Mes_*C*H_3_), 21.2 (CH(*C*H_3_)_2_), 21.1 (C_Mes_*C*H_3_) ppm. ESI-MS: [M-Cl]+= 621.1 m/z

#### 1,3-dimesityl-4-(((4-((methoxypolyethyleneglycol)amino)−4-oxobutanoyl)oxy)methyl)imidazolidin-2-yl)(2isopropoxybenzylidene)ruthenium(II)dichloride, **C3**

α-Methoxy-ω-carboxylic acid poly(ethylene glycol) (2 eq, 308 mg, 0.17 mmol) was dissolved in 2 mL dry DCM and cooled with an ice bath. EDC (3.5 eq, 56 mg, 0.29 mmol) was added and the colourless solution was stirred for 30 min. DMAP (0.4 eq, 4 mg, 0.033 mmol) was added. A second solution of **2** (54.6 mg, 0.083 mmol) in 1 mL DCM was prepared and mixed with the first solution. The reaction mixture was stirred at room temperature for 20 h and purified twice afterwards via flash chromatography with DCM and methanol. The green product was obtained as a powder after lyophilisation (141.2 mg, 68 %). ^1^H-NMR (300 MHz, CD_2_Cl_2_): δ = 16.39 (s, 1H, Ru=C*H*), 7.53 (t, 1H, H_Ar_), 7.04 (d, 4H, C_Mes_*H*), 6.90-6.80.90.80 (m, 3H, H_Ar_), 4.86 (sept, 1H, C*H*(CH_3_)_2_), 4.64 (m, 1H), 4.28 (t, 1H), 4.18 (m, 2H), 3.98 (t, 1H), 3.58 (s, 219 H, PEG), 3.47 (m, 6H), 2.40 (m, 18H), 1.2 (m, 6H) ppm. ^13^C-NMR (100 MHz, CD_2_Cl_2_): δ = 296.6 (Ru=*C*), 172.9 (*C*(O)NH), 171.1 (*C*(O)OCH_2_), 152.4 (N*C*N), 145.5 (iPr-O-*C*_Ar_), 139.4 (*C*_Mes_, C_Ar_), 130.0 (*C*_Mes_), 129.9 (*C*_Mes_, C_Ar_), 122.7 (*C*_Mes_), 122.6 (C_Ar_), 113.3 (CH-*C*_Ar_), 75.5 (NCH_2_*C*HN), 72.3 (*C*H(CH_3_)_2_), 70.9 ((*C*H_2_O)_PEG_), 70.8 ((*C*H_2_O)_PEG_), 70.7 ((*C*H_2_O)_PEG_), 70.6 (O*C*H_2_CH_2_NH), 70.1 (*C*H_2_O), 67.4 (N*C*H_2_CHN), 59.0 (O*C*H_3_), 51.9 (OCH_2_*C*H_2_NH), 39.6, 30.7 (*C*H_2_C(O)O), 28.5 (NHC(O)*C*H_2_), 21.3 (C_Mes_*C*H_3_), 21.2 (CH(*C*H_3_)_2_), 21.1 (C_Mes_*C*H_3_) ppm. MALDI-MS: [M-Cl]^+^=2417.5 *m/z*

#### (1,3-dimesityl-4-((palmitoyloxy)methyl)imidazolidin-2-yl)(2-isopropoxybenzylidene)ruthenium(II) dichloride, **C4**

A solution of **L5** (1.7 eq, 206.8 mg, 0.34 mmol) and KHMDS (2 eq, 79.4 mg, 0.4 mmol) in 4 mL dry toluene was prepared and stirred for 1 h at room temperature. A second solution of HGI (119.5 mg, 0.2 mmol) in 5 mL toluene was added to the first solution as well as Amberlyst-15. The reaction mixture was stirred at 80 °C for 18 h and purified twice via flash chromatography with hexane and ethyl acetate. A green-brown solid was acquired (83.7 mg, 47 %). ^1^H-NMR (300 MHz, CD_2_Cl_2_): δ = 16.33 (s, 1H, Ru=C*H*), 7.47 (t, 1H, H_Ar_), 7.00 (m, 4H, C*H*_Mes_), 6.86 (m, 2H, H_Ar_), 6.77 (m, 1H, H_Ar_), 4.80 (sept, 1H, C*H*(CH_3_)_2_), 4.59 (m, 1H), 4.21 (t, 1H), 4.11 (d, 2H), 3.91 (t, 1H), 2.34 (m, 16H), 2.17 (m, 4H), 1.44 (m, 1H), 1.18 (s, 30H), 0.80 (t, 3H) ppm. ^13^C-NMR (100 MHz, CD_2_Cl_2_): δ = 296.5 (Ru=*C*), 173.8 (*C*=O), 152.4 (N*C*N), 145.6 (^i^Pr-O-*C*_Ar_), 139.9 (*C*_Mes_), 139.5 (*C*_Mes_), 139.4 (*C*_Mes_), 130.0 (*C*_Mes_, C_Ar_), 122.7 (C_Ar_), 122.6 (C_Ar_), 113.4 (CH-*C*_Ar_), 75.6 (NCH_2_*C*HN& CH(CH_3_)_2_), 73.2 (CH_2_O), 63.9 (N*C*H_2_CHN), 34.3 (C(O)*C*H_2_), 32.4 (C(O)CH_2_*C*H_2_), 30.1 ((*C*H_2_)_11_), 30.0 ((*C*H_2_)_11_), 29.9 ((*C*H_2_)_11_), 29.8 ((*C*H_2_)_11_), 29.7 ((*C*H_2_)_11_), 29.5 ((*C*H_2_)_11_), 25.1 (C_Mes_*C*H_3_), 23.1 (C_Mes_*C*H_3_), 21.3 (C_Mes_*C*H_3_), 21.2 (CH(*C*H_3_)_2_), 14.3 ((CH_2_)*C*H_3_) ppm. ESI-MS: [M-Cl]^+^=859.4 *m/z* (calculated: 860.42 *m/z*).

## Olefin metathesis of phospholipids in solution and liposomes

### General procedure

Test solutions containing 2 mM lipid in methanol or liposomes in HEPES buffer (10 mM, pH 7.4) were prepared to a total volume of 750 µL. Then, 5 mol% catalyst was added (15 µL of a 5 mM stock solution in methanol) and the reaction mixture was incubated at 37 °C for 1 or 24 h. An untreated lipid solution was used as a negative control. The reaction was terminated by adding 3 eq. of imidazole and the lipids were extracted as described by Bligh and Dyer [29]. The dried extracts were dissolved in 40 µL chloroform/methanol (1:1, v/v), applied onto normal phase silica gel thin layer chromatography plates (TLC, silica gel 60, Merck), and developed with a mixture of chloroform/methanol/water (65:25:4, v/v/v). Lipids were visualized with primuline (5 mg primuline in 100 ml acetone/water, 80:20, v/v). Spots were evaluated using a digital imaging system (Biorad Chemidoc XRS Imaging System, Bio-Rad Laboratories, Munich, Germany). Image analysis was performed using Quantity One software (Bio-Rad). Experiments were typically repeated at least twice to confirm their validity, however due to slight variations these replicates were not a suitable base to calculate mean values and standard deviations. We estimate our systematic errors to be in the order of +/- 10 %, though.

### Preparative procedure

The solid phospholipid was dissolved in methanol to a concentration of 35 mM (70 µmol) and mixed with 5 mol% catalyst **HGII**. The reaction mixture was stirred at 37 °C for 24 h and analysed via TLC and ESI-MS. The products were purified via flash chromatography using silica and a gradient of 100% A to 100% B over 40 min (A: chloroform/methanol (7:3 v/v), B: chloroform/methanol/water (65:25:4 v/v/v%)). Further characterisation was carried out with ^1^H NMR spectroscopy.

### Test reactions

(i) Metathesis of different phospholipids in solution: Metathesis reactions were carried out in solution according to the general procedure using **HGII** as the catalyst and POPC, POPG, and POPE as substrates. The samples were analysed via TLC and ESI-MS. The metathesis products of POPC, POPG and POPE were identified via ^1^H NMR spectroscopy. 

 Metathesis product of POPC: ^1^H-NMR (300 MHz, CDCl_3_): δ = 5.35 (m, 2H, *H*C=C*H*), 5.18 (br, 2H, C_chiral_*H*), 4.39–4.28 (m, 5H, PO_4_-C*H*_2_CH_2_N), 4.13 (m, 2H, C*H*_2_C_chiral_HC*H*_2_), 3.93 (m, 4H, C*H*_2_C_chiral_HC*H*_2_, C*H*_2_N), 3.79 (br, 4H, C*H*_2_N), 3.66 (br, 4H), 3.35 (s, 16H, N(C*H*_3_)_3_), 2.27 (m, 7H, C*H*_2_CO_2_H), 1.96 (m, 4H, HC=CHC*H*_2_), 1.57 (br, 8H, C*H*_2_CH_2_CO_2_H), 1.25 (s, 61H, (C*H*_2_)_alkyl_), 0.87 (t, 6H, CH_2_C*H*_3_) ppm. ESI-MS: [M+H]^+^ = 1267.4 *m/z* (calculated: 1267.9 *m/z*), [M+Na]^+^ = 1289.2 *m/z* (calculated: 1289.9 *m/z*).

 Metathesis product of POPG: ESI-MS: [M-2Na]^2-^= 621.8 *m/z* (calculated: 621.4 *m/z*).

 Metathesis product of POPE: ^1^H-NMR (300 MHz, CDCl_3_): δ = 8.31 (br, 5H, CH_2_NH_3_^+^/-NH_2_), 5.35 (m, 2H, *H*C=C*H*), 5.22 (br, 2H, C_chiral_*H*), 4.38 (m, 2H, PO_4_-C*H*_2_CH_2_NH_2_), 4.35 (m, 5H, C*H*_2_C_chiral_HC*H*_2_-PO_4_-C*H*_2_), 4.15 (m, 4H, C*H*_2_C_chiral_HC*H*_2_, CH_2_NH_2_), 3.19 (br, 3H, CH_2_NH_2_), 2.31 (m, 7H, C*H*_2_CO_2_H), 1.99 (m, 4H, HC=CHC*H*_2_), 1.58 (br, 7H, C*H*_2_CH_2_CO_2_H), 1.25 (s, 60H, (C*H*_2_)_alkyl_), 0.88 (t, 6H, CH_2_C*H*_3_) ppm. ESI-MS: [M+Na]^+^ = 1205.5 *m/z* (calculated: 1205.8 *m/z*). 

 (ii) Metathesis with functionalised catalysts in solution and liposomes: The catalysts **HGII**, **C3** and **C4** were tested on the di-unsaturated lipid DOPC according to the general procedure. The metathesis products were isolated via preparative TLC and analysed via ESI-MS. See also Fig. S2.

### Metathesis on liposomes

 DOPC-based liposomes were freshly prepared by manual extrusion. Briefly, a chloroform stock of DOPC (2 μmol) was completely dried in a glass vial by rotary evaporator under vacuum (200 mbar) for at least 2 h. The lipid film was rehydrated in 1 ml HEPES buffer (10 mM, pH 7.4) at room temperature for 1 h with frequent vortexing and passed 21 times through 0.2 μm size nucleopore polycarbonate membranes mounted in a mini-extruder (Avanti Polar Lipids, Alabaster, AL). Catalysts **HGII**, **C3** and **C4** were applied to the resulting liposomes according to the general procedure.

### Quantification of ruthenium in liposomes

 A dilution series of the ruthenium standard between 0 ppb and 20 ppb with 2 % HNO_3_ was measured, and a linear calibration curve was obtained. DOPC-based liposomes (750 µL) were mixed with 15 µL of 5 mM catalyst solution in 10 mM HEPES buffer, while an untreated sample remained as a negative control. The samples were incubated at 37 °C for 4 h. Liposome-free controls were treated similarly. Both samples and controls were then separated by size exclusion chromatography (SEC). For this purpose, 2 g of Sephadex G-50 fine was swollen in 50 mL HEPES buffer (10 mM, pH 7.4) overnight at room temperature and packed into 3 mL disposable syringes with a Whatman filter at the bottom. Per column, 200 µl of sample was added and separated by centrifugation at 180 x g for 7 min. An aliquot of each unfiltered sample was retained. The ruthenium content of both filtered and unfiltered samples was determined by ICP-MS (dilution 1:10 or 1:100).

## Supplementary Information

Below is the link to the electronic supplementary material.

Supplementary Material 1

## Data Availability

The authors declare that the data supporting the findings of this study are available within the paper and its Electronic Supplementary Information files. Should any raw data files be needed in another format they are available from the corresponding author upon reasonable request.
