# Supplementary material for: Olefin metathesis of phospholipids by Ruthenium-based catalysts in solution and on liposomes under biologically relevant conditions
Source: J Biol Inorg Chem. 2025 Nov 25;30(6-8):563–75. doi: 10.1007/s00775-025-02129-6 (PMC12675672; doi:10.1007/s00775-025-02129-6)
Supplement: Supplementary file 1 — Supplementary Material 1 [file 775_2025_2129_MOESM1_ESM.pdf]

Supplementary Information

Olefin Metathesis of Phospholipids by Ruthenium-based Catalysts in Solution and on Liposomes under Biologically Relevant Conditions

Pina Eichert,[a] Huriye Deniz Uzun,[b,c] Sascha Heinrich,[d,ǂ] Thomas Günther-Pomorski,[b,c] Nils Metzler-Nolte[a,*]

[a] Faculty of Chemistry and Biochemistry, Inorganic Chemistry I – Bioinorganic Chemistry, Ruhr University Bochum, Bochum, Germany

[b] Faculty of Chemistry and Biochemistry, Molecular Biochemistry, Ruhr University Bochum, Bochum, Germany

[c] Department of Plant and Environmental Sciences, University of Copenhagen, Frederiksberg, Denmark

[d] Faculty of Chemistry and Biochemistry, Organic Chemistry I – Chemistry and Biochemistry of Natural Products, Ruhr University Bochum, Bochum, Germany

[ǂ] current address: North-Rhine Westphalian State Agency for Nature, Environment, and Consumer Protection (LANUV NRW), Duisburg, Germany

*** Corresponding author:** nils.metzler.nolte@rub.de

<u>Content:</u>

1. Initial OM experiments with different phospholipids
2. HPLC and LC-MS of self-metathesis of **4**
3. SEC and ^{102}Ru ICP-MS analysis of LUVs
4. ^{1}H NMRs of metathesis products **2** – **4**
5. Characterizing data of metathesis catalysts
6. DLS Characterization of DOPC liposomes

1. Initial OM experiments with different phospholipids in methanol

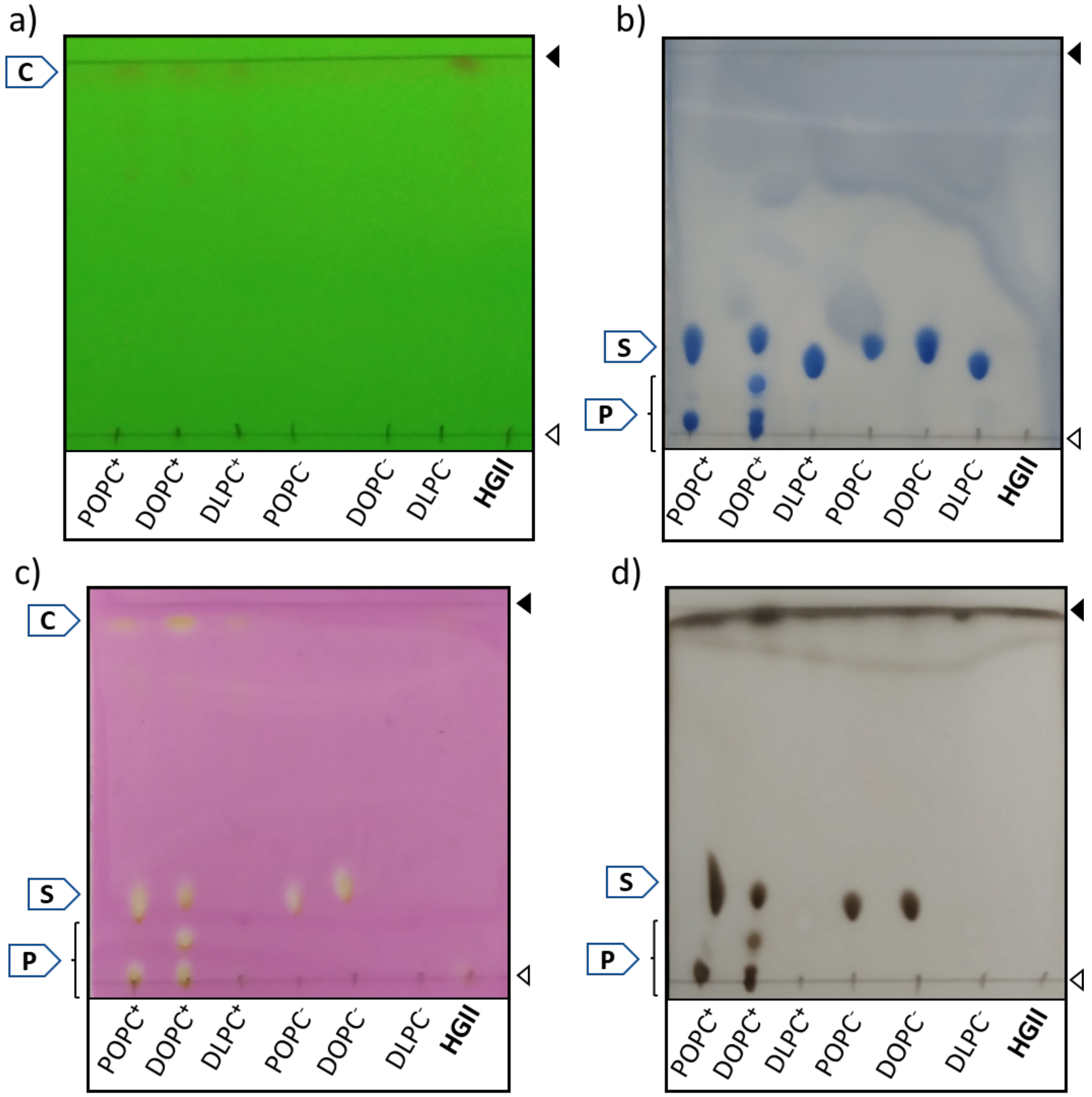

*Suppl. Figure S1: Thin-layer chromatography of initial experiments with different phospholipids using catalyst **HGII**. Individual phospholipids were treated with (+) and without (-) **HGII** in methanol and incubated for 24 h at 37 °C (preparative procedure). The TLC was carried out on a silica plate with a solvent mixture of chloroform/methanol/water (65:25:4, v/v/v). The functional groups of the lipids were visualized with fluorescence indicator F_{254} (a), molybdenum blue (b), $KMNO_4$ (c), and $CuSO_4$ (d) to image aromatic regions, phosphate groups, unsaturated moieties, and hydrocarbon chains, respectively. The di-unsaturated DOPC, mono-unsaturated POPC, and saturated DLPC were used as substrates. Open arrow, origin; filled arrow, solvent front. C: catalyst; S: substrate; P: product.*

2. HPLC and LC-MS of product **4**

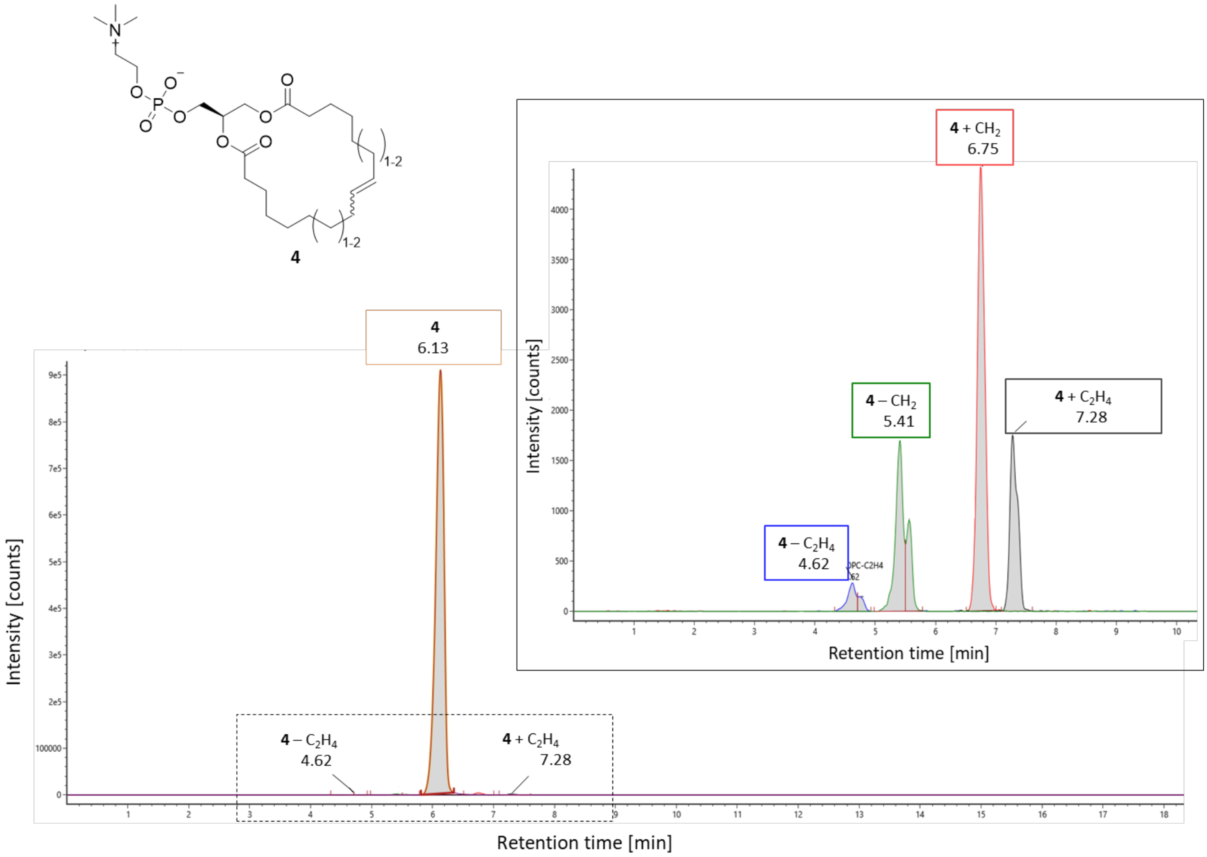

Suppl. Figure S2: LC-MS chromatogram of product ***4*** *- Self-metathesis of DOPC with* ***HGII*** *in methanol at 37 °C for 24 h yielded* ***4*** *and its derivatives from double bond migration. Measurements were carried out on a column EC 150/2 Nucleodur C18 ISIS. Mobile Phase A, H_2O, 0.1% formic acid; and B, Isopropanol, 0.1% Formic acid. Gradient: 0 – 2 min at 30% A, 2 – 8 min from 30% A to 2% A, 8 – 23 min at 2% A, 23 – 23.1 min from 2% A to 30% A and 23.1 - 25 min at 30% A with a flow rate of 0.3 mL/min at 50 °C and a detection, at 254 nm. The intervals of the retention times of the individual derivatives are labeled at the top of the chromatogram. Inset: ZOOM-In into the LC-MS chromatogram.*

Suppl.Table S1: Self-metathesis of DOPC - LC-MS of ***4*** *- overview of the detected compounds via electrospray ionization as m/z values as part of the LC-MS measurements given in Suppl. Figure S2.Neutral mass represents the calculated mass, observed mass corresponds to $[M+H]^+$ as expected.*

**Compound**	**Neutral mass (Da)**	**Observed mass (*m/z*)**
**4**	533.31	534.32
**4**+ C_2H_4	561.34	562.35
**4** + CH_2	547.33	548.33
**4** - C_2H_4	505.28	506.29
**4** – CH_2	519.30	520.30

3. SEC and ^{102}Ru ICP-MS analysis of LUVs

Suppl. Table S2: Ru loading of vesicles. DOPC liposomes were incubated with HGII, C3 and C4 and subjected to size exclusion chromatography (SEC). The ruthenium content of the elutes was determined via ICP-MS.

Sample	^{102}Ru [ppb] before SEC	^{102}Ru [ppb] after SEC	^{102}Ru [%] SEC passage
LUV	5.17	2.16	
LUV + **HGII**	3347.3	1629.1	48.7
LUV + **C4**	4972.3	213.4	4.3
LUV + **C3**	4652.6	1523.3	32.7

4. ¹H-NMRs of metathesis products **2** – **5**

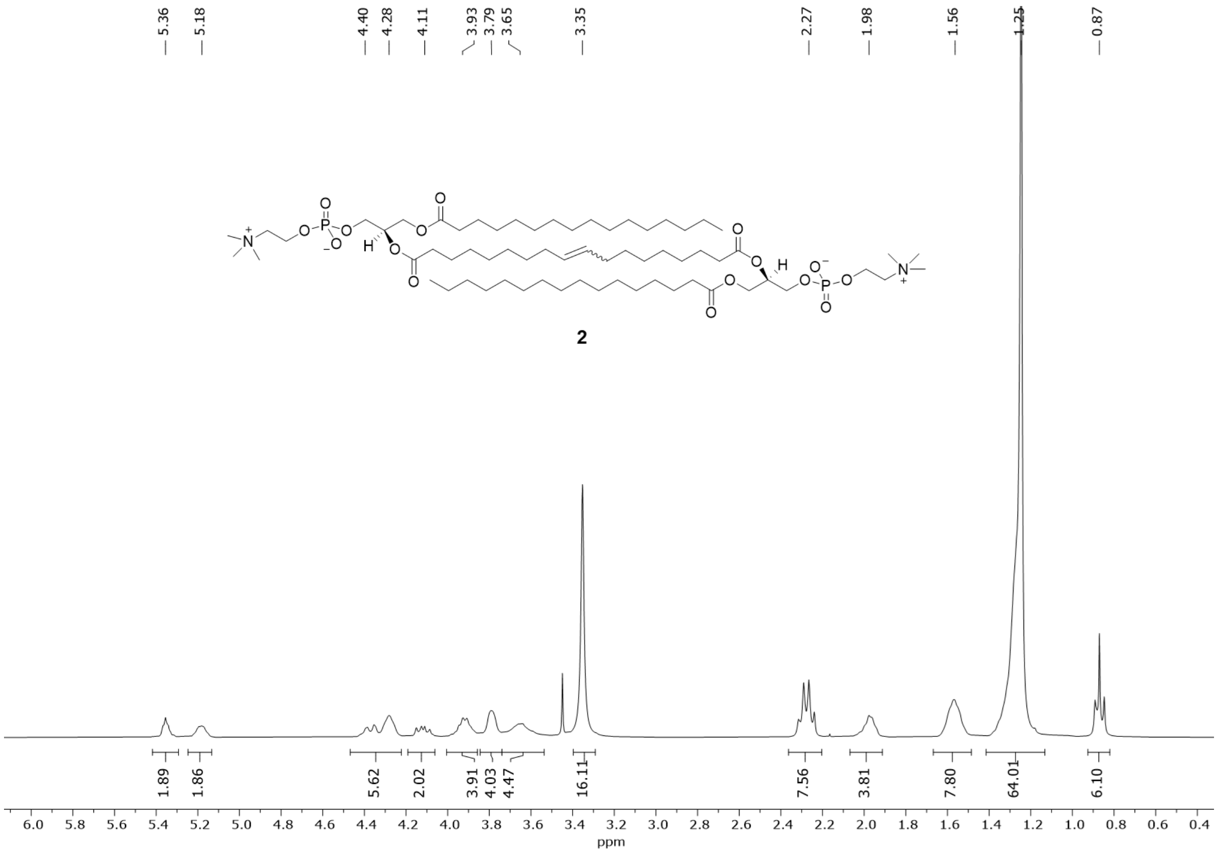

*Suppl. Figure S3: ¹H-NMR of product **2** recorded in $CDCl_3$ at 300 MHz.*

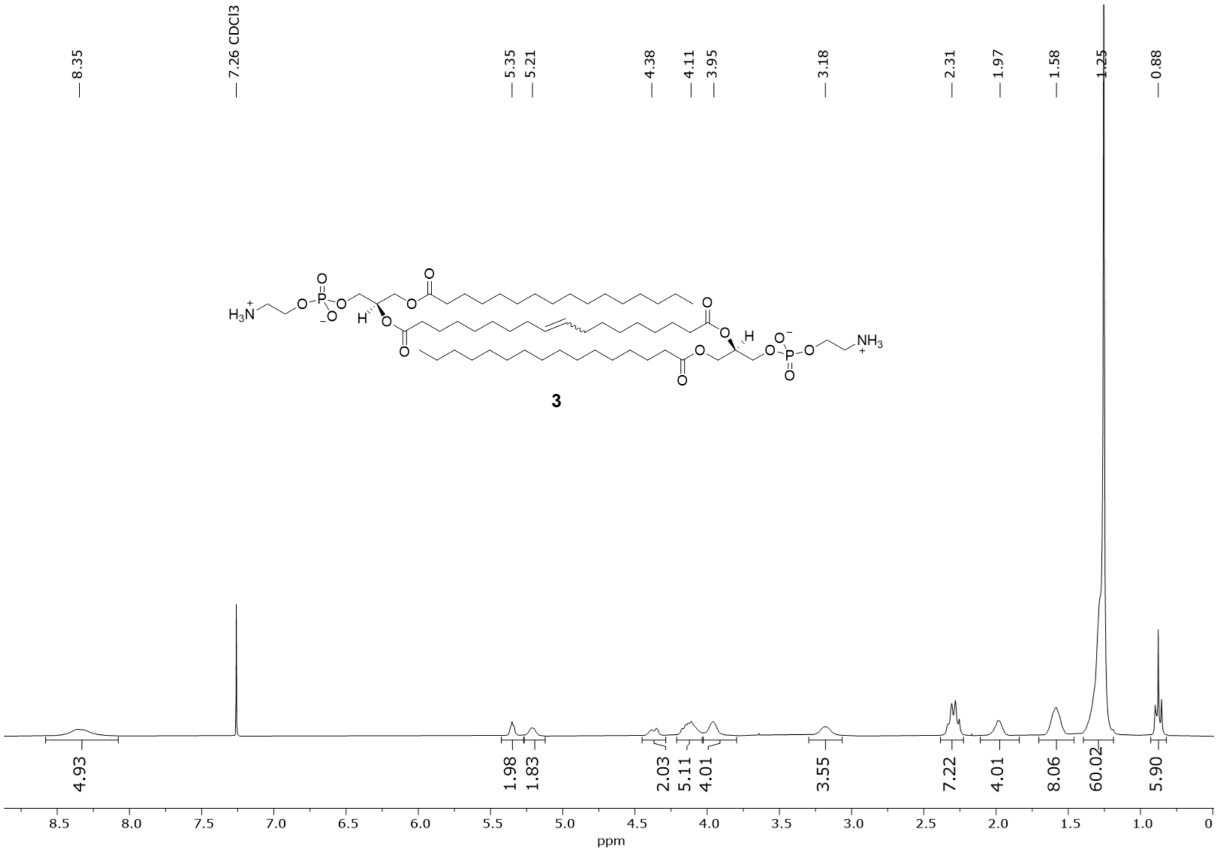

*Suppl. Figure S4: ^{1}H-NMR of **3** recorded in $CDCl_3$ at 300 MHz.*

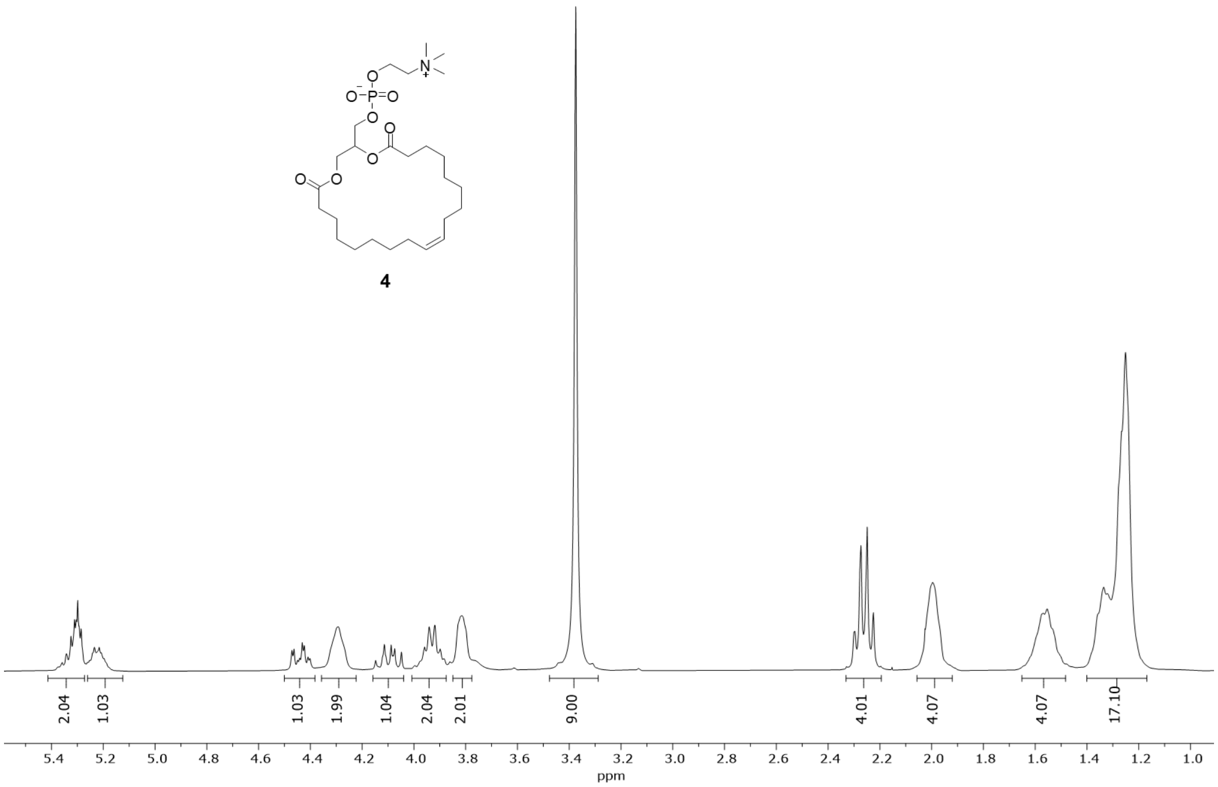

*Suppl. Figure S5: ^{1}H-NMR of **4** recorded in $CDCl_3$ at 300 MHz.*

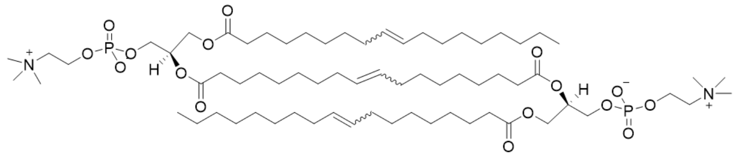

5

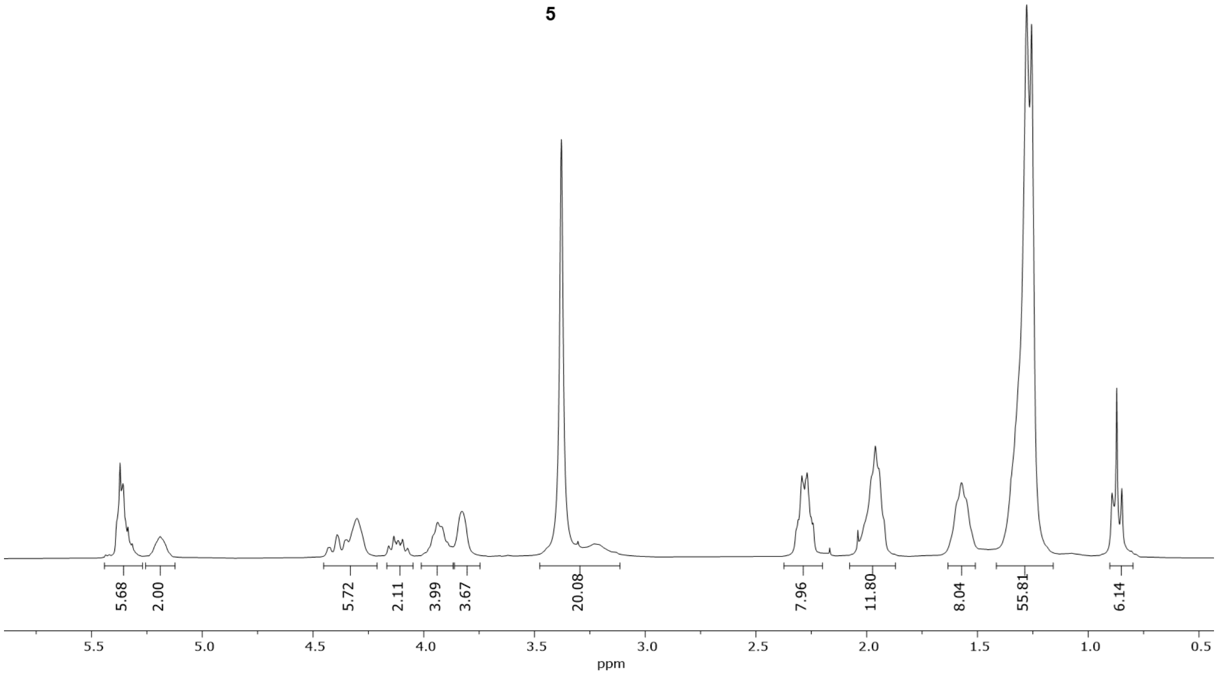

*Suppl. Figure S6: ^{1}H-NMR of **5** recorded in $CDCl_3$ at 300 MHz.*

5. Characterizing data of metathesis catalysts

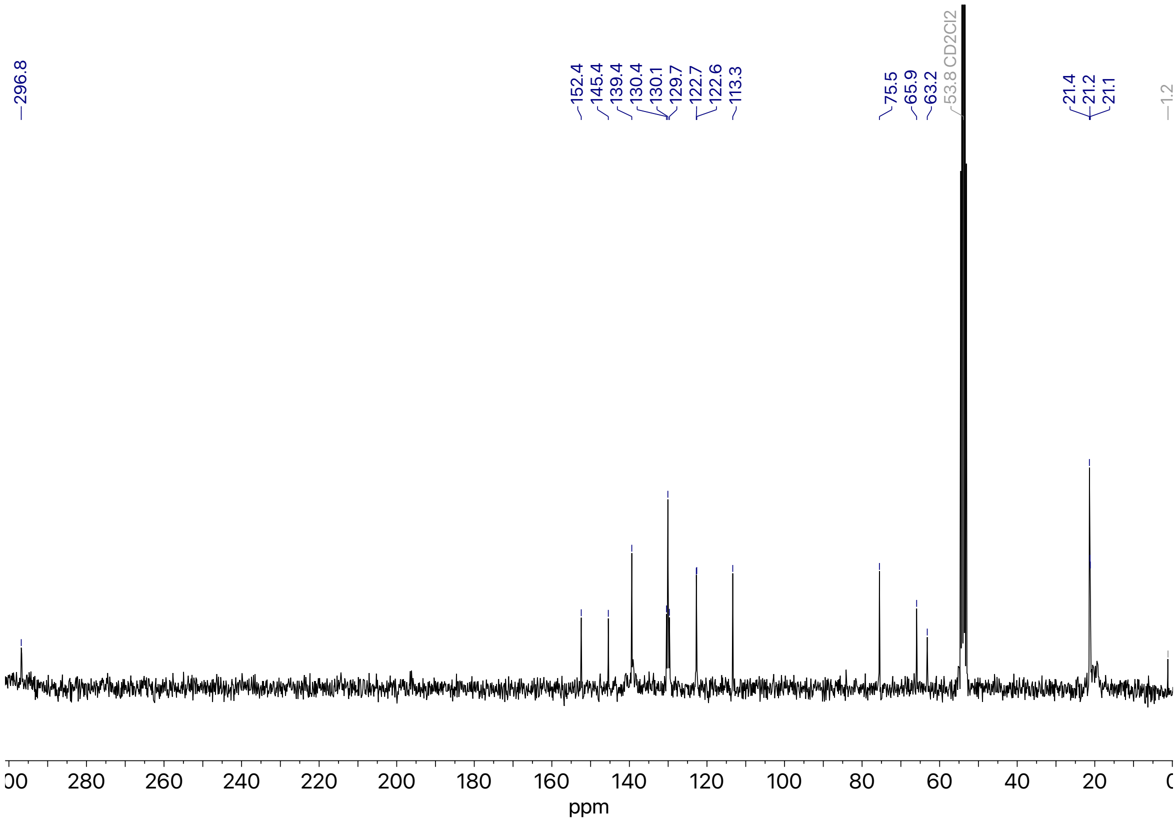

*Suppl. Figure S7: ^{13}C-NMR of **C2** in CD_2Cl_2 at 100 MHz.*

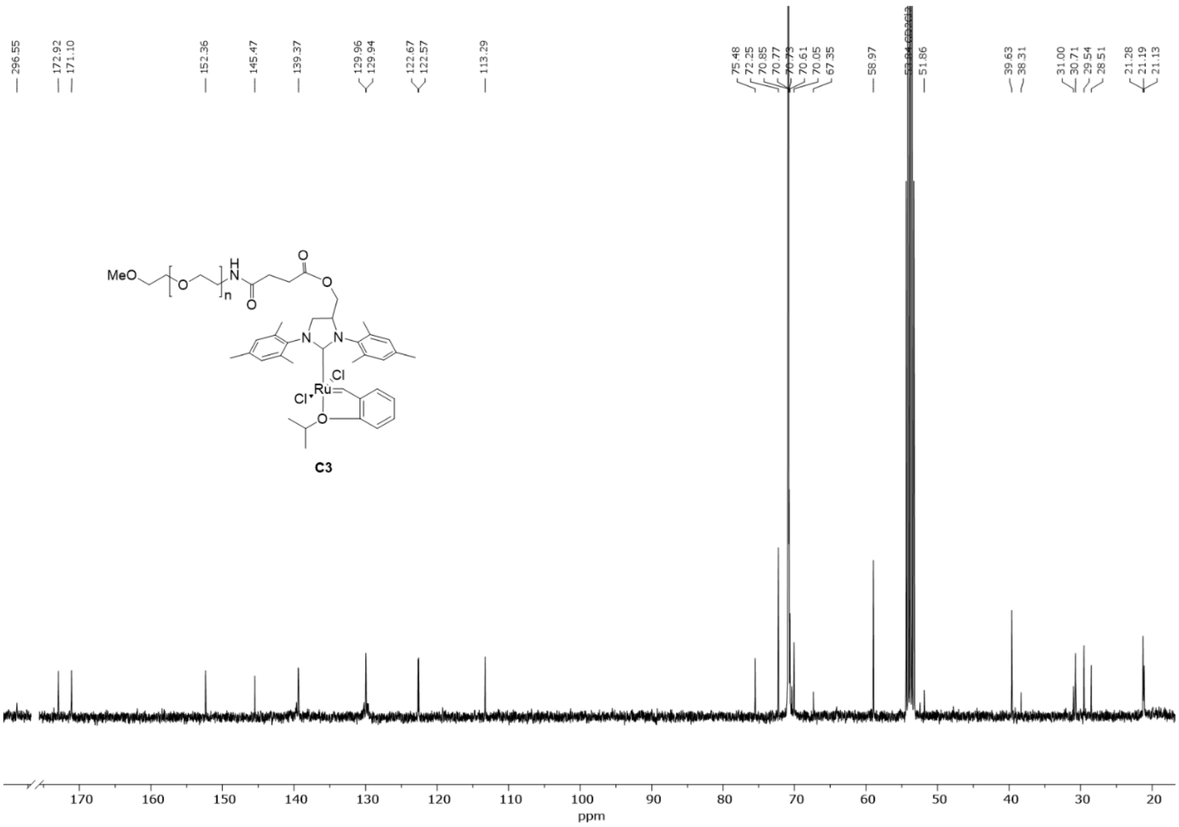

*Suppl. Figure S8: ^{13}C-NMR of **C3** in C D_2Cl_2 at 100 MHz.*

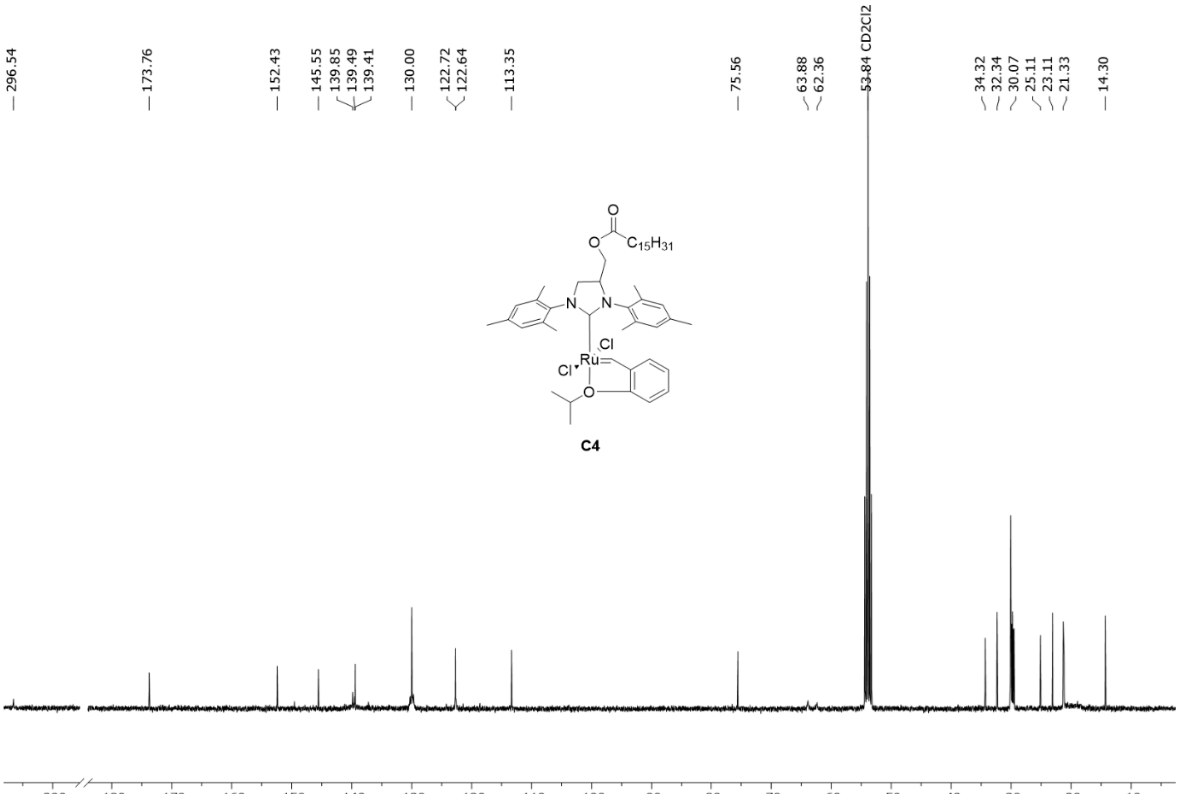

*Suppl. Figure S9: ^{13}C-NMR of **C4** in CD_2Cl_2 at 100 MHz.*

6. DLS Characterization of DOPC liposomes

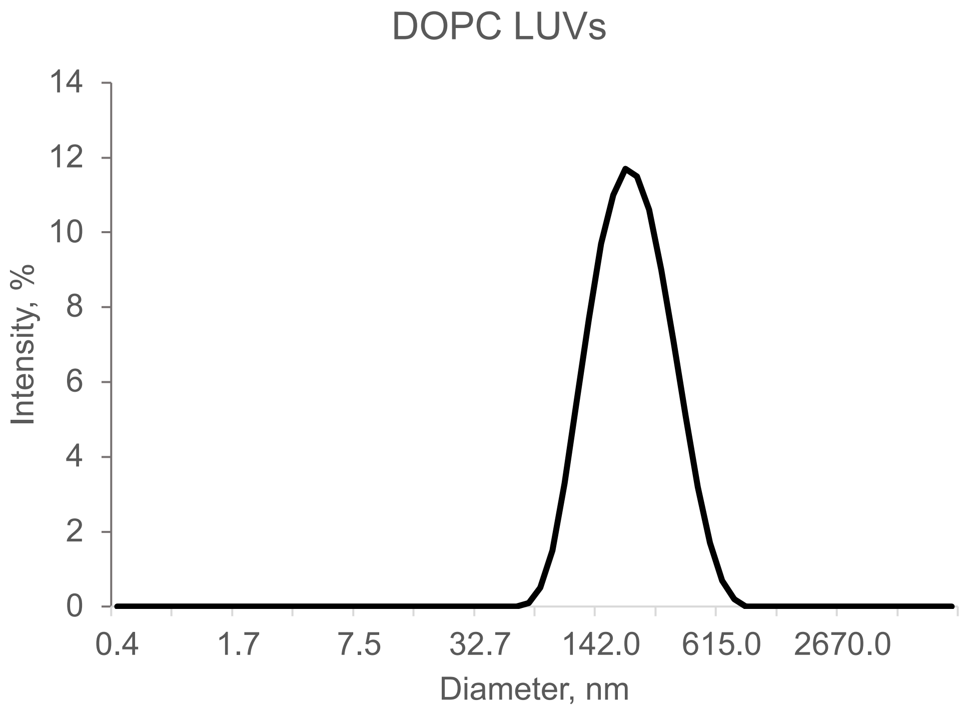

Suppl. Figure S10: ***Dynamic Light Scattering (DLS) Analysis of Large Unilamellar Vesicles (LUVs):*** *The size distribution of liposomes composed of DOPC was determined using DLS at 25 °C with a Zetasizer Nano ZS instrument (Malvern, Worcestershire, UK). Each sample was measured in ten technical replicates. The average vesicle diameter was 226 ± 4.8 nm, with a polydispersity index (PdI) of 0.177.*
